# The American Dental Association

**Published:** 1886-06

**Authors:** J. N. Crouse

**Affiliations:** Chairman of Committee of Arrangements, 2231 Prairie Avenue, Chicago, Ill.


THE AMERICAN DENTAL ASSOCIATION.—The Committee of Arrangements had hoped to give full and definite information in the July number of Journals in regard to all the details of the arrangements for the annual meeting of the Association to be held at Niagara Falls, August 3rd. But as the railroad rates thus far secured have not been as satisfactory as the Committee yet hope to secure and are working for, they will issue a circular later to all members of the Association and to local societies as far as possible. Those who are not members of the Association and who wish the circular will please drop a postal to the Chairman of the Committee to insure their getting it. The railroad rates as thus far secured on all leading lines are one and a third fare round trip to be issued upon presentation of certificate. Definite information concerning this will be given in the circular if better terms and arrangements are not secured. The hotel rates will be as follows: The International Hotel will receive dentists and their families at $3.00 per day; the Cataract at $4.00; the Niagara, Prospect Park and Hotel Atlantique $2.00 per day if rooms are applied for and secured in advance. The Park Theatre, adjoining the International Hotel has been secured as the place of meeting. Do not be anxious about not receiving the circular; you will get it sometime in July, but a few days delay in issuing the circular may mean a good deal of money saved to those attending the Association. For instance, the arrangements were not completed and circular issued until latter part of July last year. A month earlier it would have been *impossible* to have gotten the low rates finally secured. Remember, we *promise* nothing better than we now publish but will continue to work for more favorable terms.

All State and local Societies which have adopted substantially the Code of Ethics of the American Dental Association, will remember that they are entitled to one delegate for every five members. Such delegates must have credentials signed by the President and Secretary of the Society which they represent.

J. N. CROUSE,
*Chairman of Committee of Arrangements,*
2231 Prairie Avenue, Chicago, Ill.

